# The Prayer Cure Practically Applied

**Published:** 1887-04

**Authors:** 


### THE PRAYER CURE PRACTICALLY APPLIED.

Among the members of the Board of New York Aldermen of 1884–5, who have been indicted and held for trial for receiving bribes, is Thomas Cleary, who, if we mistake not, is a member in good standing of St. Peter's Roman Catholic Church, whose parochial schools number some twelve hundred pupils of all grades. It is said that Alderman Cleary, in the days of his high-feathered prosperity, was a liberal contributor to the support of these schools, on account of which, he is still held in no common esteem by those who benefited by his charities, which appear to have been earned with so little mental effort or strain upon his conscience.

Whether this church-going church, supporting Aldermen obtained in the old-fashioned way of his church, anything in the nature of an indulgence, covering this little matter of a $20,000 bribe, more or less, we are left to conjecture. At all events, the little army of twelve hundred was moved as by a single impulse, to marshall its forces and try the effect of the *prayer cure* upon this almost hopeless case of moral obliquity, but not so far as we have been able to learn, out of any concern for the *reformation* of the damaged Alderman, but solely and simply for his escape from the just punishment of his alleged offences. It is in the nature of an appeal to the higher court before trial, to prevent the trial itself, or at all events a conviction. Little children are not apt, out of their own volition, to give heed to such matters, and there were, doubtless, some older heads that set on foot this rather novel enterprise; but just how it was to be made available, we are not informed. Perhaps, it was anticipated that any court and jury so obdurate as to insist upon following the letter of the law, by proceeding to try the offender, like Saul of Tarsus, would be overwhelmed by Divine interposition, before accomplishing their purpose.

But what should be said of the almost criminal absurdity of leading the minds of little children into such reprehensible ways of thinking and believing. What trust will they be likely to place in their prayerful supplications to the Throne of Grace, when they learn that their heart-offerings have been rejected, and the prison gates have closed upon him upon whom they have been taught to look as their benefactor? Let the directors of this unprecedented prayer-cure effort answer this pertinent inquiry, and remember, hereafter, that ours is "a government of the people, by the people, and for the people," and that one of the safeguards against crime is the certainty of punishment which should always overtake the offender.

The foregoing is not the only example of the invocation of Divine interference in the settlement of legal differences. In a late judicial contest, at New Orleans, involving the validity of a will bequeathing the snug little sum of two million dollars out of the celebrated Gains estate, it transpired that the *quasi* legatee undertook to carry her point by bargaining with the priest for the prayers of his sanctimonious flock, offering to *come down handsomely* in case of success, but the one witness, also a Catholic, who was relied upon to carry this point under heavenly inspiration, was refractory enough to "tell the truth and shame the devil," as the old proverb has it, and so the whole gang of church machinery was run to waste and the spurious parchment relegated to the waste-paper basket.

But there is a very serious side to this sort of profanity, which the most obtuse of vision cannot fail to see, and we venture the opinion that the great body of Catholics in this country would be no less severe in their condemnation of it, than the good and true of all other denominational creeds and orders of belief. It is only the ignorant and fanatical that resort to measures which savor so strongly of blasphemy. The spirit which it evinces, is not only immoral, but dangerous, and cannot be too severely reprehended. To instill youthful minds with a belief in the efficacy of prayers which can only be answered at the expense of justice, is to so poison the soul and corrupt the understanding as to unfit them for citizenship, especially in a free community like ours, where the theory that justice ever holds her even scales, is not altogether a fiction, although it does sometimes seem as if the sublime goddess managed to see just a little, with one eye; but this is, doubtless, a prejudice, which prevails to some extent outside of politica rings.

